# Our Portrait Gallery

**Published:** 1884-05

**Authors:** 


### OUR PORTRAIT GALLERY.

We purpose giving to our readers, from time to time, through THE JOURNAL, portraits of some of the more prominent members of our profession, giving short sketches of the lives of each as their portraits appear.

In this issue we give an exact likeness of one of the most distinguished members of the profession in Georgia,

### DR. ABNER WELLBORN CALHOUN.

Dr. Calhoun was born in Newnan, Georgia, April 16th, 1846. Here he resided until the outbreak of the war in 1861, when he entered the army—a mere boy, only fifteen years old—with the First Georgia Regiment, and remained constantly with it until the close of the war, when he returned to his native town and began the study of medicine.

He graduated from Jefferson Medical College in the spring of 1869, and returned to Newnan where he practiced medicine until the spring of 1871, when he went to Germany and continued his studies in Berlin and Vienna for three years.

In the fall of 1873 while he was still in Europe, he was elected to the chair of Diseases of the Eye, Ear and Throat, in the Atlanta Medical College, a position he still fills and honors.

In 1874 he was elected a member of the Medical Association of Georgia, and in 1883 was elected President of the Association. He is now President, his term of office expiring in April, 1885.

No man in Georgia has risen more rapidly than Dr. Calhoun. He is an honor to the profession and to the State.

